# O‐GlcNAcylation Mediates Glucose‐Induced Alterations in Endothelial Cell Phenotype in Human Diabetes Mellitus

**DOI:** 10.1161/JAHA.119.014046

**Published:** 2020-06-06

**Authors:** Nobuyuki Masaki, Bihua Feng, Rosa Bretón‐Romero, Elica Inagaki, Robert M. Weisbrod, Jessica L. Fetterman, Naomi M. Hamburg

**Affiliations:** ^1^ The Whitaker Cardiovascular Institute Department of Medicine Boston University School of Medicine Boston MA

**Keywords:** diabetes mellitus, endothelial nitric oxide synthase, insulin resistance, O‐GlcNAc, Vascular Biology, Metabolism

## Abstract

**Background:**

Posttranslational protein modification with O‐linked *N*‐acetylglucosamine (O‐GlcNAc) is linked to high glucose levels in type 2 diabetes mellitus (T2DM) and may alter cellular function. We sought to elucidate the involvement of O‐GlcNAc modification in endothelial dysfunction in patients with T2DM.

**Methods and Results:**

Freshly isolated endothelial cells obtained by J‐wire biopsy from a forearm vein of patients with T2DM (n=18) was compared with controls (n=10). Endothelial O‐GlcNAc levels were 1.8‐ford higher in T2DM patients than in nondiabetic controls (*P*=0.003). Higher endothelial O‐GlcNAc levels correlated with serum fasting blood glucose level (*r*=0.433, *P*=0.024) and hemoglobin A_1c_ (*r*=0.418, *P*=0.042). In endothelial cells from patients with T2DM, normal glucose conditions (24 hours at 5 mmol/L) lowered O‐GlcNAc levels and restored insulin‐mediated activation of endothelial nitric oxide synthase, whereas high glucose conditions (30 mmol/L) maintained both O‐GlcNAc levels and impaired insulin action. Treatment of endothelial cells with Thiamet G, an O‐GlcNAcase inhibitor, increased O‐GlcNAc levels and blunted the improvement of insulin‐mediated endothelial nitric oxide synthase phosphorylation by glucose normalization.

**Conclusions:**

Taken together, our findings indicate a role for O‐GlcNAc modification in the dynamic, glucose‐induced impairment of endothelial nitric oxide synthase activation in endothelial cells from patients with T2DM. O‐GlcNAc protein modification may be a treatment target for vascular dysfunction in T2DM.

**Nonstandard Abbreviations and Acronyms**
Akt: protein kinase B
eNOS: endothelial nitric oxide synthase
HAECs: human aortic endothelial cells
HbA_1c_: hemoglobin A_1c_
HG: high‐glucose concentration
NG: normal glucose concentration
OGA: O‐GlcNAcase
O‐GlcNAc: O‐linked N‐acetylglucosamine
OGT: O‐GlcNAc transferase
PI3K: phosphatidylinositol 3‐kinase
T2DM: type 2 diabetes mellitus

**Clinical Perspective**

**What Is New?**

- Using freshly isolated endothelial cells harvested from patients with type 2 diabetic mellitus, the protein modification O‐linked *N*‐acetylglucosamine was found to be elevated by immunofluorescent microscopy.
- The O‐linked *N*‐acetylglucosamine levels in the freshly isolated endothelial cells correlated with serum levels of glucose and hemoglobin A_1c_.
- Modulation of intracellular O‐linked *N*‐acetylglucosamine levels in the freshly isolated endothelial cells changes the ability of insulin‐induced endothelial nitric oxide synthase activation.

**What Are the Clinical Implications?**

- Regulation of O‐linked *N*‐acetylglucosamine is a potential mediator of reversible endothelial dysfunction in human with type 2 diabetic mellitus.

Patients with type 2 diabetes mellitus (T2DM) have an excess of cardiovascular events and premature vascular aging characterized by endothelial dysfunction. Altered nutrient levels of T2DM, including elevated glucose, lead to an increase in irreversible glycosylation of hemoglobin. Hemoglobin A_1c_ (HbA_1c_) is a widespread, useful biomarker of chronic circulating glucose levels in clinical practice. However, recent works have emphasized the importance of intracellular adhesion with O‐linked *N*‐acetylglucosamine (O‐GlcNAc), called O‐GlcNAcylation, which is known as a posttranslational modification.

O‐GlcNAc is a byproduct of the hexosamine biosynthesis pathway, a minor portion of glycolysis.^1^ Approximately 1% to 3% of fructose‐6 phosphate converted from incoming glucose enters the hexosamine biosynthesis pathway.^2^ Uridine diphosphate GlcNAc is the end product of the hexosamine biosynthesis pathway and a precursor of glyco‐conjugates including hyaluronan.^3^ In addition, O‐GlcNAc transferase mediates the adhesion of uridine diphosphate GlcNAc to serine and threonine residues of proteins in the nucleus and in cytosol by O‐link that can be reversed by O‐GlcNAcase (OGA). O‐GlcNAc modification regulates cellular functions through alterations in signaling, epigenetics, and gene expressions.^4^, ^5^, ^6^, ^7^ It is known that O‐GlcNAc modification is essential for mammalian embryogenesis.^8^, ^9^

The roles of O‐GlcNAc modification in the cardiovascular system have been investigated. O‐GlcNAc increases in relation to high cellular glucose uptake and oxidative stress.^10^, ^11^, ^12^ Acutely, O‐GlcNAc modification is thought to play a protective role for cell survival against external stress‐inducing inflammation.^13^, ^14^ However, persistent elevation of intracellular O‐GlcNAc levels can cause chronic diseases including diabetic angiopathy.^15^ In T2DM, impaired activation of the insulin receptor/insulin receptor substrate/PI3K (phosphatidylinositol 3‐kinase)/Akt (protein kinase B)/endothelial nitric oxide synthase (eNOS) pathway is involved in endothelial dysfunction because insulin contributes to maintenance of chronic vasodilation via NO synthesis.^16^, ^17^, ^18^, ^19^ Prior experimental studies suggest that excessive O‐GlcNAc modification that occurred in proteins of the insulin receptor/insulin receptor substrate/PI3K/Akt/eNOS pathway can reduce NO production in endothelium.^20^ Hyperglycemia and hexosamine activation decrease phosphorylation of eNOS by Akt.^21^ Specifically, O‐GlcNAc competitively inhibits the binding site of eNOS phosphorylation by Akt.^22^

However, the evidence linking O‐GlcNAc to the vasculature in human T2DM is limited. We previously identified an adverse endothelial cell phenotype characterized by endothelial insulin resistance and lower NO production in patients with T2DM.^23^, ^24^, ^25^, ^26^, ^27^ The present study investigates the association of O‐GlcNAc with glucose levels and eNOS signaling in freshly isolated endothelial cells from patients with T2DM. Our hypothesis is that modulation of intracellular O‐GlcNAc levels changes the ability of insulin‐induced phosphorylation of eNOS at serine 1177, an activated form of eNOS for NO production. O‐GlcNAc may be a mediator of endothelial dysfunction of T2DM, especially as an early and reversible cause for its enzymatic regulation.

## Methods

The data that support the findings of this study are available from the corresponding author on reasonable request. Additional methods and results can be found in Data S1.

### Study Participants

Adult patients with T2DM (defined as fasting serum glucose ≥126 mg/dL or ongoing treatment for T2DM) and nondiabetic participants (fasting glucose <100 mg/dL) were enrolled in this study. A blood examination was performed in the fasting state in the morning at the Boston Medical Center Clinical Laboratory. All participants stopped vasoactive medications on the morning of the study. The study protocol was approved by the Boston Medical Center institutional review board, and all participants provided written informed consent.

### Peripheral Endothelial Cell Collection

The procedure for peripheral venous endothelial cell biopsy has been described previously.^24^, ^25^, ^26^ Briefly, a 20‐gauge intravenous catheter was inserted into a superficial forearm vein under sterile technique. A 0.018‐in J‐wire (Arrow International) was introduced through the catheter, and endothelial cells were taken by gentle abrasion of the vessel wall. In the laboratory, endothelial cells were removed from the wire tip by centrifugation in a dissociation buffer and plated on poly‐l‐lysine–coated microscope slides (Sigma‐Aldrich). The retrieved cells were treated or cultured as described below or fixed immediately in 4% paraformaldehyde. Slides were washed in PBS, dried, and stored at −80°C until further processing.

### Culture of Freshly Isolated Endothelial Cells

The endothelial cells harvested from patients were plated on 16‐well Chamber slides (Thermo Scientific) after coating with fibronectin. We subsequently incubated the cells in endothelial growth medium‐2 Bullet Kit medium (Lonza Inc) and 10% FBS with either high‐glucose (HG) concentration (30 mmol/L) or normal glucose (NG) concentration (5 mmol/L) for 24 hours. The endothelial cells were cultured with a highly selective OGA inhibitor, Thiamet G (Cayman Chemical), 1 μmol/L for 24 hours or a nonspecific O‐GlcNAc transferase inhibitor (Alloxan; Sigma‐Aldrich) 2.5 mmol/L for 24 hours. Thiamet G was dissolved in dimethyl sulfoxide at a stock solution of 80 mmol/L and further diluted in medium at a concentration <0.1%. The vehicle was the same dose of dimethyl sulfoxide. The medium was replaced with albumin and growth factor–free medium 3 hours before fixation with 4% paraformaldehyde without changing the concentrations of glucose and Thiamet G. Insulin stimulation (10 nmol/L) was performed 30 minutes before fixation.

### Culture of Commercially Available Human Aortic Endothelial Cells

Commercially available cultured human aortic endothelial cells (HAECs; Lonza Inc), passages 4 to 7 grown to 80% of confluence, were used for all experiments. The cells were incubated in NG or HG for 24 to 48 hours. The cells were treated with or without Thiamet G 0.01 to 5.0 μmol/L for 24 hours. For immunoblotting, the cells were serum starved for 3 hours before collection without changing concentrations of glucose and Thiamet G. The cells were removed from the culture dish with a cell lysis buffer (Cell Signaling) containing 1% protease inhibitor cocktail (Sigma‐Aldrich). Cell lysates were spun at 20 000*g* for 15 minutes at 4°C. The supernatants were stored at −80°C. Protein concentration was determined by the Pierce BCA protein assay (Thermo Fisher Scientific).

### Western Blot Analysis

Proteins were subjected to 8% to 20% gradient gels of SDS‐PAGE (GE Healthcare) or 4% to 15% gradient gels (Invitrogen) and transferred to polyvinylidene difluoride membranes. Membranes were blocked (PBS, 0.1% Tween 20, 5% bovine serum albumin) for 1 hour. Membranes were probed in blocking buffer containing primary antibodies of 1:1000 dilution: phosphorylated eNOS at serine 1177 (BD Biosciences), followed by the appropriate horseradish peroxidase–conjugated secondary antibody. Immunoreactions were visualized with Amersham ECL Plus Western Blotting Detection Reagents (GE Healthcare). Membranes were stripped (62.5 mmol/L Tris‐HCl [pH 6.8], 100 mmol/L mercaptoethanol, 2% SDS) for 30 minutes at 50°C and reprobed with O‐GlcNAc (RL2; Abcam), eNOS/NOS type III, antiactin, or glyceraldehyde‐3‐phosphate dehydrogenase antibodies of 1:1000 dilution to verify equal protein loading. The bands were quantified by densitometry (LAS‐3000 mini; FUJIFILM).

### Assessment of Protein Expression by Quantitative Immunofluorescence

Fixed sample slides were thawed and rehydrated with PBS containing 50 mmol/L glycine (Sigma‐Aldrich) for 10 minutes. The cells on the slides were permeabilized with 0.1% Triton X‐100, and nonspecific binding sites were blocked with 0.5% BSA. The slides were incubated overnight at 4°C with primary antibodies against the following targets: O‐GlcNAc (RL2; 1:200 dilution; Abcam), phosphorylated eNOS at serine 1177 (1:200 dilution; Abcam). All slides were double‐stained with an anti–von Willebrand factor antibody (1:300 dilution; Dako) for identification of endothelial cells. The slides were washed with 50 mmol/L glycine and incubated for 1 hour at 37°C with corresponding Alexa Fluor 488 and Alexa Fluor 594 antibodies (1:200 dilution; Invitrogen). The slides were washed again with 50 mmol/L glycine, and glass cover slips were mounted with Vectashield containing 4′,6‐diamidino‐2‐phenylindole for nuclear identification (Vector Laboratories). For each batch of patient‐derived cells, we stained a control slide of cultured HAECs, maintained in endothelial growth medium‐2 Bullet Kit medium (Lonza) at 37°C with 5% CO_2_ and taken from a single index passage.

The quantification of immunofluorescent intensity was described previously.^26^ Slide images of a fluorescence microscope (Nikon Eclipse TE2000‐E) at ×20 were captured using a Photometric CoolSnap HQ2 Camera (Photometrics). Exposure time was constant, and image intensity was corrected for background fluorescence. Fluorescent intensity was quantified by NIS Elements AR Software (Nikon Instruments). For each protein of interest, fluorescent intensity was quantified in 20 cells from each patient, per condition, and averaged. Intensity is expressed in arbitrary units, which is the percentage of the average fluorescence intensity from the patient sample to the average fluorescence intensity of the HAEC slide stained at the same time. This formula is for adjusting the deviation of staining condition. The quantification was performed blinded to participant identities, and each batch included patients with and without diabetes mellitus.

### Statistical Analysis

Statistical analyses were performed using SPSS v22.0 (IBM Corp). The distribution of continuous clinical characteristics and measurements were evaluated by examining a histogram and Shapiro–Wilk test. The independent samples *t* test or Mann–Whitney *U* test was used for 2‐group comparison, as appropriate. Categorical clinical characteristics were compared using χ^2^ testing. The correlation coefficient between endothelial O‐GlcNAc levels and fasting blood glucose or HbA_1c_ levels was obtained with the Pearson method because these variables were normally distributed.

In ex vivo experiments using freshly isolated endothelial cells, we used a nonparametric method for the small sample size. The Wilcoxon signed rank test was used for comparing paired samples of freshly isolated endothelial cells from the same patient to assess treatment effects. The Kruskal–Wallis test was used for multitreatment comparisons in immunoblotting with commercially available cultured HAECs. Post hoc comparison between 2 groups was performed by the Mann–Whitney *U* test. All laboratory data were expressed as mean±SD. The data of experimental studies were expressed as mean±SE. A 2‐sided *P*<0.05 was considered statistically significant.

## Results

### Study Participants

Endothelial cells were collected from 31 of patients with T2DM and 10 nondiabetic controls for this study. The group of T2DM patients tended to be older and included higher percentages of African ethnicity and women. None of the nondiabetic controls had a medical history of hypertension or dyslipidemia (Table). The patients with T2DM had higher body mass index and blood triglyceride concentration.

**Table 1:** Clinical Characteristics

Characteristic	Controls (n=10)	Participants With T2DM (n=31)	*P* Value
Age, y	49±9	55±8	0.070
Female sex	3 (30)	18 (58)	0.119
Ethnicity			0.052
White	6 (60)	5 (16)	
Black	4 (40)	23 (74)	
Hispanic	0	1 (3)	
Asian	0	2 (7)	
Body mass index	28±4	36±10	0.014
Hypertension	0	20 (65)	<0.001
Dyslipidemia	0	18 (58)	<0.001
Smoking ever	4 (40)	20 (65)	0.159
Total cholesterol, mg/dL	184±26	190±39	0.724
LDL‐C, mg/dL	118±23	114±36	0.710
HDL‐C, mg/dL	52±10	46±14	0.268
Triglycerides, mg/dL	71±16	150±77	0.005
HbA_1c_ , %	5.4±0.3	7.5±2.0	0.007
Fasting glucose, mg/dL	79±9	153±81	0.007
Medication			
DPP4 inhibitor	0	1 (3)	
SU	0	6 (19)	
Metformin	0	20 (65)	
Insulin	0	6 (19)	

Data are expressed as mean±SD or n (%), as appropriate. DPP‐4, dipeptidyl peptidase 4; HbA_1c_, hemoglobin A_1c_; HDL‐C, high‐density lipoprotein cholesterol; LDL‐C, low‐density lipoprotein cholesterol; SU, sulfonylureas; T2DM, type 2 diabetes mellitus.

### Association of Endothelial O‐GlcNAc Levels With T2DM and Glucose Measures

**Figure 1:**
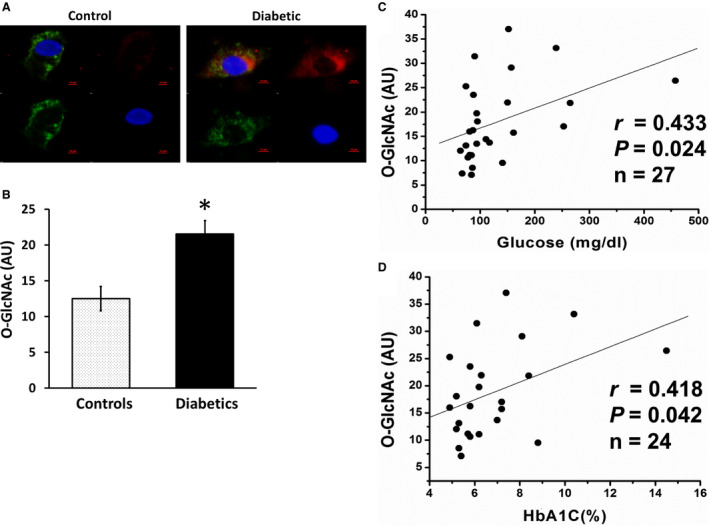
Increased intracellular adhesion with O‐linked *N*‐acetylglucosamine (O‐GlcNAc; called O‐GlcNAcylation) in endothelial cells from patients with type 2 diabetes mellitus (T2DM). **A** and **B**, O‐GlcNAc protein modification levels were evaluated by an antibody against O‐GlcNAc (red); 4′,6‐diamidino‐2‐phenylindole (blue); von Willebrand factor (green). Representative endothelial cell from a patient with T2DM (right; n=18) shows higher O‐GlcNAc level compared with a representative cell from a nondiabetic control (left; n=10). The summed data of both groups show that higher fasting glucose (**C**) and higher hemoglobin A_1c_ (HbA_1c_; **D**) levels were associated with higher O‐GlcNAc levels in endothelial cells. Data are expressed as mean±SE. *P=0.003. AU indicates arbitrary units.

Overall levels of O‐GlcNAc were higher in endothelial cells isolated from patients with T2DM (n=18) than from nondiabetic controls (n=10, *P*=0.003; Figure 1A and 1B). Higher blood glucose levels (*r*=0.433, n=27, *P*=0.024) and higher HbA_1c_ (*r*=0.418, n=24, *P*=0.042) were both associated with higher endothelial cell O‐GlcNAc levels (Figure 1C and 1D). There was a trend for an association of higher body mass index with higher endothelial O‐GlcNAc levels (*r*=0.343, n=28, *P*=0.074). There was no association of O‐GlcNAc levels with age, low‐density lipoprotein cholesterol, high‐density lipoprotein cholesterol, or triglycerides. The results suggest that hyperglycemia is a driver for higher endothelial O‐GlcNAc modifications.

### Reversibility of Excess Endothelial Cell O‐GlcNAc Modification and Restoration of Insulin‐Mediated eNOS Activation by NG in T2DM

**Figure 2:**
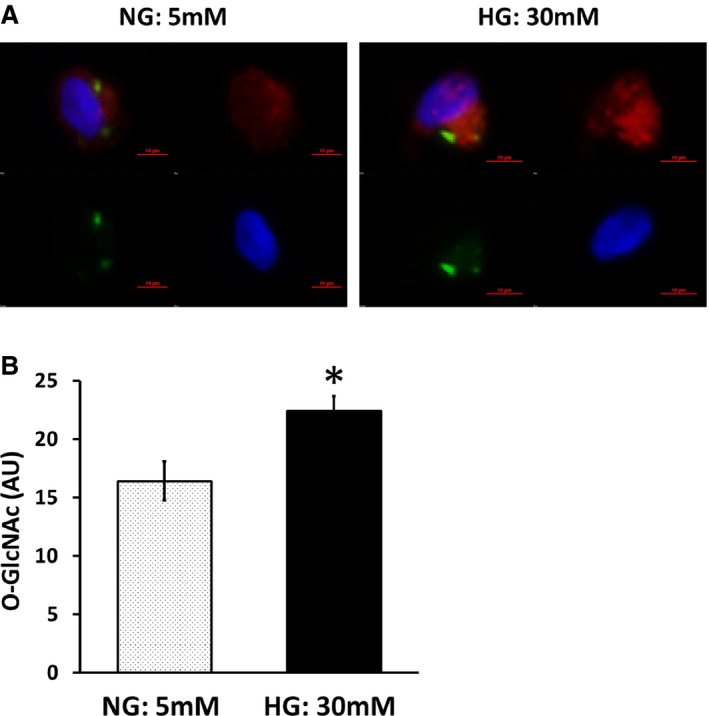
Normal glucose (NG) reduces endothelial O‐linked *N*‐acetylglucosamine (O‐GlcNAc) levels in patients with type 2 diabetes mellitus (T2DM). Venous endothelial cells were isolated from patients with T2DM, and O‐GlcNAc levels were measured after 24 hours of incubation in high glucose (HG) or NG conditions. **A**, O‐GlcNAc levels were studied in endothelial cells from patients with T2DM after HG (30 mmol/L) or NG (5 mmol/L). Representative cells from the same patient are shown. **B**, Pooled data show that O‐GlcNAc levels decreased after NG treatment (n=9, *P=0.011). Data are expressed as mean±SE. AU indicates arbitrary units.

We investigated whether the elevated O‐GlcNAc modification in endothelial cells from patients with T2DM patients was reversible by altering glucose conditions. Endothelial cells isolated from patients with T2DM were incubated in either HG (30 mmol/L) or NG (5 mmol/L) for 24 hours. O‐GlcNAc intensity was reduced in NG conditions compared with HG conditions (n=9, *P*=0.011; Figure 2A and 2B), indicating that O‐GlcNAc modification is rapidly reversible by restoring normal glucose conditions.

**Figure 3:**
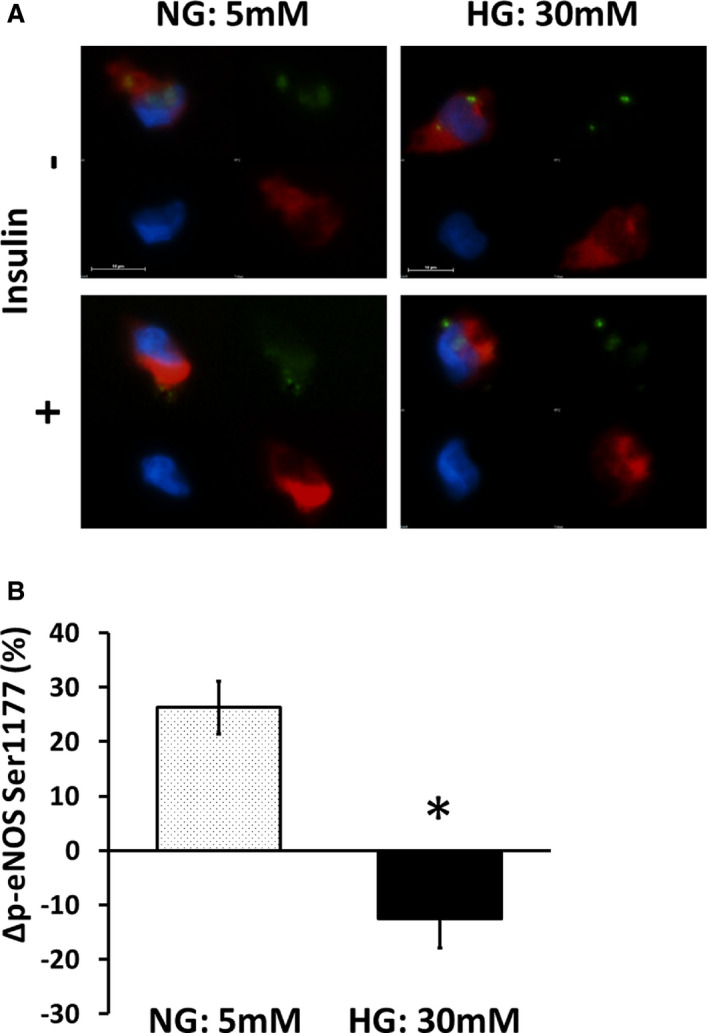
Normal glucose (NG) restores endothelial function in endothelial cells from patients with type 2 diabetes mellitus (T2DM). Freshly isolated endothelial cells from patients with T2DM were incubated in high glucose (HG; 30 mmol/L) or NG (5 mmol/L) for 24 hours and then treated with 0 or 10 nM insulin for 30 minutes and then fixed. **A**, Endothelial nitric oxide synthase (eNOS) activation was evaluated as eNOS phosphorylation at serine 1177 (Ser1177). Representative cells from the same patient are shown. Red, phosphorylated eNOS (p‐eNOS); green, von Willebrand factor; blue, 4′,6‐diamidino‐2‐phenylindole. **B**, Pooled data demonstrated that NG restores insulin‐induced eNOS phosphorylation in endothelial cells isolated from patients with T2DM (n=10, *P=0.005). Data are expressed as mean±SE. AU indicates arbitrary units.

We demonstrated previously that venous and arterial endothelial cells isolated from patients with T2DM show insulin resistance characterized by reduced insulin‐mediated eNOS phosphorylation.^24^, ^26^, ^27^ However, whether the abnormal endothelial signaling in T2DM is reversible is not known. As shown in Figure 3, endothelial cells isolated from patients with T2DM incubated in NG conditions for 24 hours had restored insulin‐mediated eNOS phosphorylation compared with those incubated in HG conditions (n=10, *P*=0.005). These findings suggest that the abnormal endothelial phenotype in T2DM is reversible with exposure to NG conditions.

**Figure 4:**
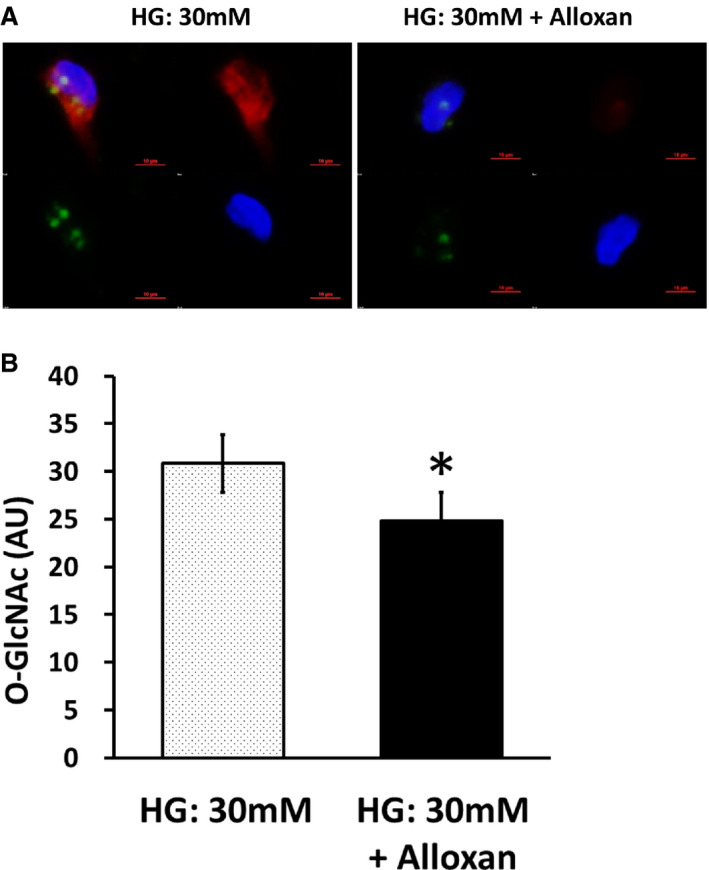
O‐linked *N*‐acetylglucosamine (O‐GlcNAc) transferase inhibition reduces endothelial O‐GlcNAc levels in patients with type 2 diabetes mellitus (T2DM). Venous endothelial cells were isolated from patients with T2DM, and O‐GlcNAc levels were measured after 24 hours of incubation in high glucose (HG) condition (30 mmol/L) with or without Alloxan, an O‐GlcNAc transferase inhibitor. **A**, O‐GlcNAc levels of representative cells from the same patient are shown. Red, O‐GlcNAc; green, von Willebrand factor; blue, 4′,6‐diamidino‐2‐phenylindole. **B**, Pooled data show that O‐GlcNAc levels decreased after Alloxan treatment (n=6, *P=0.028). Data are expressed as mean±SE. AU indicates arbitrary units.

The O‐GlcNAc intensity was also decreased by Alloxan (n=6, *P*=0.028; Figure 4), which is an O‐GlcNAc transferase inhibitor but is known as a diabetogenic compound for toxicity through reactive oxygen species generation, and further examination was not performed.^28^

### Maintenance of High O‐GlcNAc Levels Impairs Insulin‐Mediated eNOS Activation in T2DM

**Figure 5:**
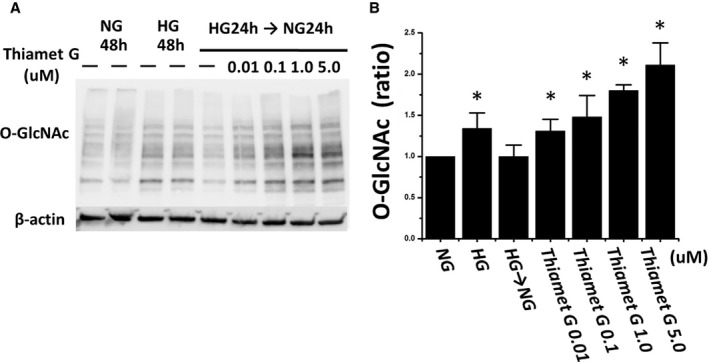
Dose‐dependent increase of O‐linked *N*‐acetylglucosamine (O‐GlcNAc) by O‐GlcNAcase inhibition with Thiamet G in human aortic endothelial cells (HAECs). Commercially available cultured HAECs were incubated with normal glucose (NG) medium (5 mmol/L) or high glucose (HG) medium (30 mmol/L) for 48 hours (n=4). We also incubated HAECs in HG for 24 hours and replaced by NG for the next 24 hours. Thiamet G was added in the changed NG medium at the concentration of 0 to 5 μmol/L. The immunoblotting image shows that O‐GlcNAc levels increase with increasing concentrations of Thiamet G (**A**). Quantization of O‐GlcNAc levels on the reference of NG for 48 hours are shown (**B**). Data are expressed as mean±SE. *P<0.05 vs NG for 48 hours.

**Figure 6:**
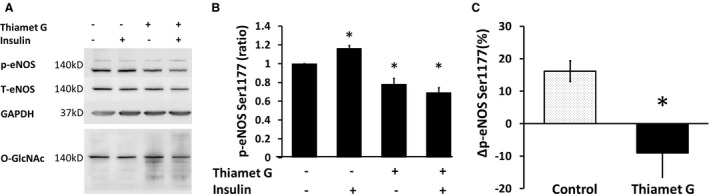
Inhibition of O‐linked *N*‐acetylglucosamine‐ase (O‐GlcNAcase) with Thiamet G inhibits insulin‐stimulated endothelial nitric oxide synthase (eNOS) activation in human aortic endothelial cells (HAECs). Commercially available cultured HAECs were incubated in normal glucose (NG) medium (5 mmol/L) with or without Thiamet G 1 μmol/L for 24 hours and stimulated by insulin 0 or 10 nmol/L for 30 minutes before collection. The images of immunoblotting are phosphorylated eNOS (p‐eNOS), total eNOS, GAPDH, and O‐GlcNAc which was subsequently stained after total eNOS (**A**). The bar graph shows summed data on the reference of the no‐treatment group (**B**). The percentage increase of p‐eNOS by insulin stimulation was significantly reduced by Thiamet G (**C**; n=6, *P=0.046). Ser1177 indicates serine 1177.

Our findings with NG conditions suggest a link between glucose‐mediated O‐GlcNAc modifications and impaired insulin‐mediated eNOS activation. To further support a link between changes in O‐GlcNAc levels and insulin signaling, we investigated whether high O‐GlcNAc levels induced under NG conditions was sufficient to maintain endothelial cell insulin resistance. To evaluate the appropriate dosing for Thiamet G—an inhibitor of OGA, the protein responsible for degrading O‐GlcNAc—we cultured commercially available HAECs in NG, HG, and NG with escalating concentrations of Thiamet G. HG increased O‐GlcNAc levels that were lowered by NG levels and increased by Thiamet G (Figure 5). Treatment of HAECs under NG conditions with Thiamet G 1 μmol/L for 24 hours impaired insulin‐mediated eNOS phosphorylation (n=6, *P*=0.001; Figure 6A and 6B). The percentage increase of phosphorylated eNOS was significantly reduced by Thiamet G (n=6, *P*=0.046; Figure 6C), recapitulating the phenotype observed in the endothelial cells from patients with T2DM.

**Figure 7:**
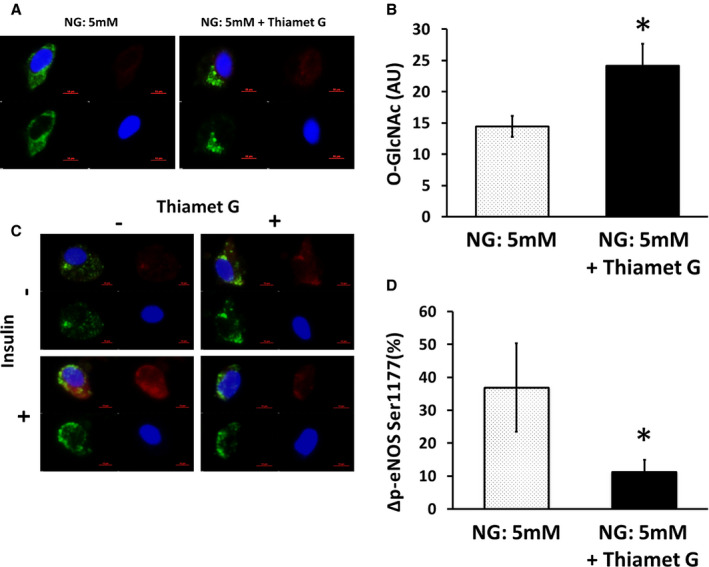
Maintenance of elevated O‐linked *N*‐acetylglucosamine (O‐GlcNAc) levels prevents restoration of insulin‐mediated endothelial nitric oxide synthase (eNOS) activation in patients with type 2 diabetes mellitus (T2DM). Venous endothelial cells were isolated from patients with T2DM, and O‐GlcNAc levels were measured after 24 hours of incubation in normal glucose (NG; 5 mmol/L) conditions with or without 1 µmol/L of Thiamet G. **A**, Representative cells from the same patient are shown. Red, O‐GlcNAc; green, von Willebrand factor (vWF); blue, 4′,6‐diamidino‐2‐phenylindole (DAPI). **B**, Pooled data show that O‐GlcNAc levels were higher in the presence of Thiamet G (n=6, *P=0.028). Then, venous endothelial cells were isolated from patients with T2DM and incubated in NG medium (5 mmol/L) with or without Thiamet G 1 µmol/L for 24 hours and stimulated by insulin 0 or 10 nmol/L for 30 minutes before collection and fixation. **C**, Representative cells from the same patient are shown. Red, phosphorylated eNOS; green, vWF; blue, DAPI. **D**, Pooled data demonstrated that Thiamet G prevented the restoration of insulin‐induced eNOS phosphorylation by NG in endothelial cells isolated from patients with T2DM (n=8, *P*=0.017). Data are expressed as mean±SE. AU indicates arbitrary units.

Endothelial cells from patients with T2DM were incubated for 24 hours in NG medium or NG plus Thiamet G 1 μmol/L. Compared with NG alone, Thiamet G increased endothelial O‐GlcNAc levels (n=6, *P*=0.028; Figure 7A and 7B). Compared with NG conditions, endothelial cells treated with NG with Thiamet G had impaired insulin‐stimulated eNOS activation (n=8, *P*=0.017; Figure 7C and 7D).

In addition, we evaluated eNOS activation by NO levels using 5,6‐diaminofluorescein diacetate fluorescence in the arterial endothelial cells from nondiabetic patients treated by Thiamet G (Table S1). The increase of insulin‐mediated 5,6‐diaminofluorescein diacetate intensities was lower in the cells treated by Thiamet G compared with the vehicle treated control cells (Figure S1). Thiamet G increased intracellular O‐GlcNAc levels 1.4‐fold in the condition of the experiment (Figure S2).

## Discussion

The findings of the this study support a link between elevated glucose with protein modification using O‐GlcNAc and abnormal endothelial phenotype in patients with T2DM. We show that endothelial cell O‐GlcNAc levels are increased in patients with T2DM and are associated with both glucose and HbA_1c_. The endothelial insulin resistance observed in patients with T2DM is reversible with exposure to NG conditions along with reduction in endothelial O‐GlcNAc levels. Maintenance of elevated O‐GlcNAc levels with an OGA inhibitor led to persistent endothelial insulin resistance even under NG conditions. Taken together, these findings indicate that O‐GlcNAc modification mediates the adverse consequences of HG on endothelial cell insulin responsiveness.

Prior studies support a link between human T2DM and elevated O‐GlcNAc modification in several cell types. O‐GlcNAc levels were higher in erythrocytes,^29^, ^30^ leukocytes,^31^ and whole blood^32^ from patients with T2DM. The endothelium is exposed to excess circuiting glucose in a similar fashion to blood cells; therefore, endothelial cells are a likely target of elevated O‐GlcNAc modification that may alter endothelial function. O‐GlcNAc was abundant in the endothelial layer of carotid arteries in patients with T2DM, consistent with our findings in venous endothelial cells.^21^ In addition, visceral adipose tissue samples from hyperglycemic patients showed increased levels of O‐GlcNAc–modified proteins with increased reactive oxygen species generation and decreased OGA activity. These data indicate that O‐GlcNAcylation contributes to the decrease of adipocyte‐derived NO release.^33^ In addition to the human studies, previous studies with animal models or cultured endothelial cells demonstrated that O‐GlcNAc modification can also be increased by aging,^34^ hypoxia,^35^ and hypertension.^36^

Our study extends the prior human studies by connecting elevated O‐GlcNAc levels directly to high glucose and impaired endothelial responses to insulin. Studies in experimental animal models and cell culture support a contribution of O‐GlcNAc to endothelial function.^37^, ^38^, ^39^, ^40^, ^41^ In cultured endothelial cells, inhibition of glutamine:fructose‐6‐phosphate amidotransferase, the rate‐limiting enzyme of the hexosamine biosynthesis pathway, reversed suppressed eNOS phosphorylation by hyperglycemia.^22^ The inhibition of glutamine:fructose‐6‐phosphate amidotransferase by azaserine restored endothelium‐dependent arterial dilation in myograph.^38^ Endothelium‐specific OGA overexpression resulted in improvement of vascular relaxation in a type 1 diabetic mouse model.^39^ These findings suggest that inhibition of O‐GlcNAc leads to restoration of endothelial dysfunction caused by hyperglycemia.

Furthermore, PugNAc, an OGA inhibitor, increased reactivity to constrictor stimuli and decreased acetylcholine‐induced vascular relaxation that was accompanied by decreased levels of phosphorylated eNOS at serine 1177 and phosphorylated Akt at serine 473.^40^ This phenomenon is partially explained by the negative effect of O‐GlcNAc on the IL‐10 (interleukin 10)/JAK1 (Janus kinase 1)/STAT3 (signal transducer and activator of transcription 3) pathway, with increased ERK1/2 (extracellular signal–regulated kinase 1/2) activity.^41^ Thus, our findings provide direct evidence in humans with T2DM that O‐GlcNAc modification relates to reversible endothelial dysfunction, consistent with previous studies.

In contrast, the concept of diabetic memory has been proposed to explain why diabetic angiopathy continues to progress even after appropriate control of fasting blood glucose levels.^42^, ^43^, ^44^ The role of O‐GlcNAc for diabetic memory has not been fully elucidated. O‐GlcNAc modification of SP1 (Sp1 transcription factor) in endothelial cells upregulates PAI1 (plasminogen activator inhibitor 1),^45^ ICAM1 (intracellular adhesion molecule 1),^46^ and ROBO4 (roundabout guidance receptor 4),^47^ which are involved in the angiogenesis and integrity of arteries. MicroRNA miR‐200a/200b plays a protective role in HG‐induced endothelial inflammation by lowering O‐GlcNAc transferase expression, and these microRNAs are decreased in T2DM.^48^ Thus, there may be aspects of endothelial dysfunction attributable to O‐GlcNAc that may not be so readily improved with restoration of normoglycemia. Further investigation with freshly isolated endothelial cells is expected.

This study has several limitations. First, the number of endothelial cells derived from J‐wire abrasion is small. The method used for quantitation is limited to a single immunoblotting technique. In addition, we were not able to specifically assess the degree of eNOS modification with O‐GlcNAc in endothelial cells from patients with T2DM because the number of cells obtained was too small to permit immunoprecipitation of eNOS alone. Second, we studied venous endothelial cells instead of arterial endothelial cells, given the accessibility in a larger group of patients. Prior studies have suggested similar phenotypes between arterial and venous endothelial cells harvested from patients.

Our findings suggest that O‐GlcNAc protein modification is a dynamic regulator of the endothelial cell phenotype in patients with T2DM in response to glucose conditions. Our findings may have clinical relevance to the acute and chronic effects of glucose on vascular function and suggest that modulation of O‐GlcNAc could have a favorable impact on vascular dysfunction in human diabetes mellitus.

## Sources of Funding

This project was supported by National Heart, Lung, and Blood Institute grants HL081587, HL115391, and HL102299. Fetterman is supported by KO1 HL143142, and Breton‐Romero is supported by AHA SDG. Masaki is supported by a Grant‐in‐aid for Scientific Research from the Ministry of Education, Science, and Culture of Japan (17K09565).

## Disclosures

None.

## Supporting information


**Data S1**
**Table S1**
**Figures S1–S2**
**References** 24, 27
