# Supplementary material for: O‐GlcNAcylation Mediates Glucose‐Induced Alterations in Endothelial Cell Phenotype in Human Diabetes Mellitus
Source: J Am Heart Assoc. 2020 Jun 6;9(12):e014046. doi: 10.1161/JAHA.119.014046 (PMC7429031; doi:10.1161/JAHA.119.014046)
Supplement: Supplementary file 1 — Data S1 Table S1 Figures S1–S2 References 24, 27 [file JAH3-9-e014046-s001.pdf]

# SUPPLEMENTAL MATERIAL

**Data S1.**

## Introduction

We evaluated eNOS activation by determining eNOS phosphorylation at Ser1177. The supplemental material is sought to determine NO levels by 5,6-diaminofluorescein diacetate (DAF-2 DA) fluorescence in the endothelial cells from non-diabetic patients treated by Thiamet G, a high-specific O-GlcNAcase (OGA) inhibitor. This would support a functional correlation between O-GlcNAc and eNOS activity.

## Materials and Methods

### Subjects

Patients who underwent elective cardiac catheterization from November 2019 to January 2020 at the National Defense Medical College Tokorozawa, Japan were enrolled in the supplemental study. Exclusion criteria included ongoing treatment for diabetes mellitus, malignant tumor and hemodialysis.

The study protocol was approved by the National Defense Medical College Review Board, and all participants provided written informed consent. The details of diagnostic criteria, laboratory examinations were shown in our previous study.[27]

**Endothelial cells**

Fresh arterial endothelial cells were harvested from patients without diabetes mellitus as described previously.[27] Briefly, an inner dilator of a radial catheter sheath used for coronary angiography was extracted under a sterile technique. In the laboratory, endothelial cells adherent to the disposable device were removed by washing with an erythrocyte lysing kit (R&D Systems) and the pellet was obtained by centrifugation. The pellet was washed again with endothelial basal medium (EBM-2; Lonza, Basel, Switzerland) and plated on poly-L-lysine-coated microscope slides (Sigma, St. Louis, MO).

**Nitric oxide detection**

Endothelial nitric oxide levels were visualized and quantified using DAF-2 DA staining, a membrane-permeable fluorescent indicator for nitric oxide by a

modified previous method.[24] Endothelial cells were incubated with Thiamet G (Cayman Chemical) 1 μmol/L, or vehicle in endothelial growth medium-2 Bullet Kit medium (Lonza Inc.) and 10% FBS with normal glucose concentration (NG: 5 mmol/L) for 8 hours. The cells were loaded with DAF-2 DA fluorescence probe (10 μmol/L, Goryo chemical, Sapporo, Japan) in quiescent medium with Thiamet G or vehicle at 37°C for 30 minutes in darkness followed by insulin stimulation (100 nmol/L, 30 minutes). The slides were fixed with 4% paraformaldehyde and double-stained with an anti–von Willebrand Factor (vWF) antibody (1:300 dilution; Invitrogen, Carlsbad, CA) at 4°C for 16 hours for identification of endothelial cell. The slides were washed with 50 mmol/L glycine twice and incubated for 2 hours at 37°C with corresponding Alexa Fluor-594 antibodies (1:200 dilution; Invitrogen). The slides were washed and mounted under glass coverslips with Vectashield containing 4′,6 - diamidino - 2 - phenylindole (DAPI) for nuclear identification (Vector Laboratories, Burlingame, CA).

Slide images of a fluorescence microscope at × 40 magnification were captured (KEYENCE, Osaka, Japan). Exposure time was constant, and image intensity was corrected for background fluorescence. Fluorescent intensity was

quantified by a software (KEYENCE, Osaka, Japan) in 20 cells from each slide and averaged. Wilcoxon Signed Rank test was used for comparing paired samples of freshly isolated endothelial cells from a same patient to assess treatment effects. The data of experimental studies were expressed as mean ± standard error. A 2-sided P value < 0.05 was considered statistically significant.

**Assessment of O-GlcNAc modification by Quantitative Immunofluorescence**

Freshly isolated endothelial cells were treated with or without Thiamet G in chamber slides for 8 hours and preserved in a refrigerator at -80°C after fixing with 4% paraformaldehyde. In another day, the slides were incubated overnight at 4°C with primary antibodies against O-GlcNAc (RL2) (1:50 dilution; Santa-Cruz, Dallas, TX). The slides were double-stained with anti–von Willebrand Factor (vWF) antibody (1:300 dilution; Thermo Fisher Scientific) for identification of endothelial cells. After incubation, the slides were washed and incubated for 3 h at 37 °C with corresponding Alexa Fluor-488 and Alexa Fluor-594 antibodies (1:200 dilution; Invitrogen, Carlsbad, CA). The detail of immunofluorescence

microscope was described previously.[24,27]

## Results

The freshly isolated arterial endothelial cells taken from patients were incubated with or without Thiamet G 1 μM for 8 hours. The characteristics of the patients were shown in **Table S1**. The increase of insulin-mediated DAF-2 intensities was lower in the cells treated by Thiamet G than the controls (**Figure S1**). We also confirmed that intracellular O-GlcNAc levels were increased (1.4-fold) by Thiamet G (**Figure S2**).

**Table S1. Clinical Characteristics.**

| Characteristic | Patients (n = 8) |
|---|---|
| Age (yrs.) | 69 ± 12 |
| Sex, n (women, %) | 3 (38) |
| Ethnicity, n (%) | |
| Asian | 8 (100) |
| Body mass index | 23 ± 3 |
| CAD, n (%) | 3 (38) |
| Hypertension, n (%) | 2 (25) |
| Dyslipidemia, n (%) | 2 (25) |
| Smoking ever, n (%) | 2 (25) |
| LDL cholesterol (mg/dL) | 100 ± 32 |
| HDL cholesterol (mg/dL) | 63 ± 16 |
| Triglycerides (mg/dL) | 124 ± 53 |
| HbA1C (%) | 6.3 ± 0.9 |
| Fasting glucose (mg/dL) | 97 ± 7 |

Data are expressed as means ± standard deviation or count (%) as appropriate.

CAD, coronary artery disease, LDL, low-density lipoprotein; HDL, high-density lipoprotein.

HbA1C; Hemoglobin A1C

**Figure S1. Inhibition of OGA with Thiamet G attenuates insulin-stimulated NO production.**

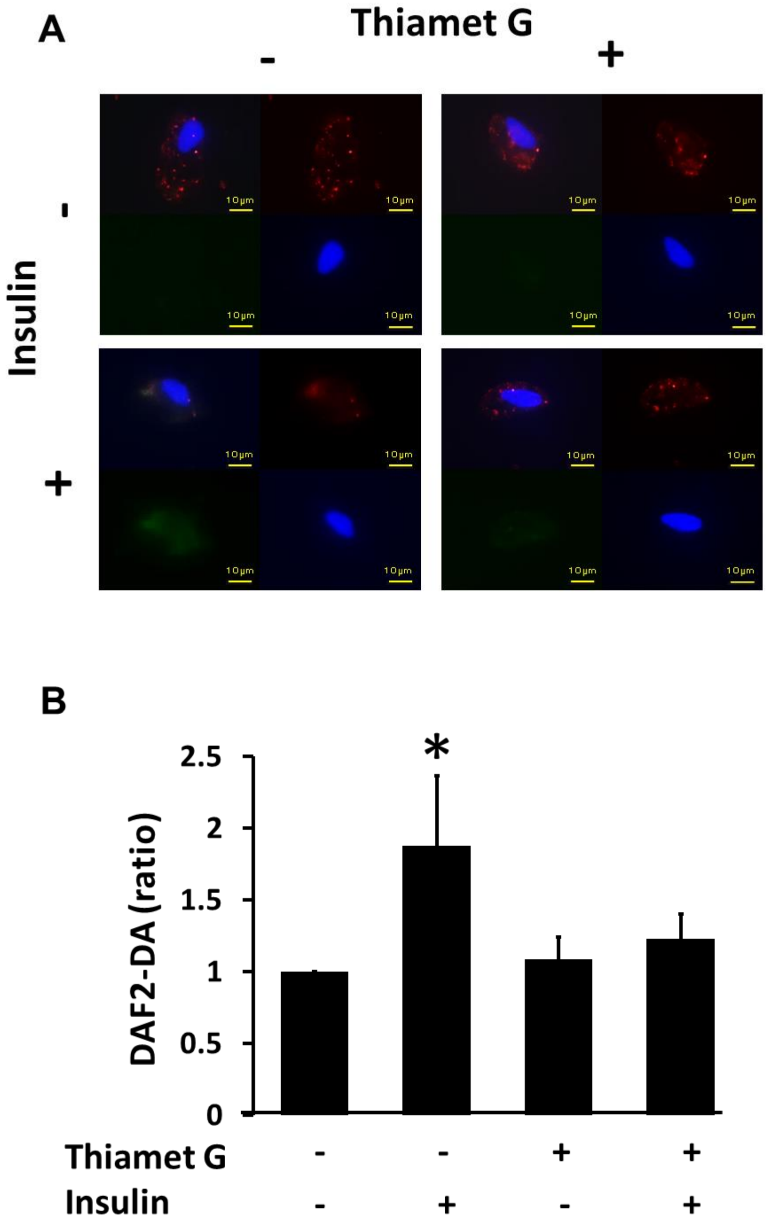

C

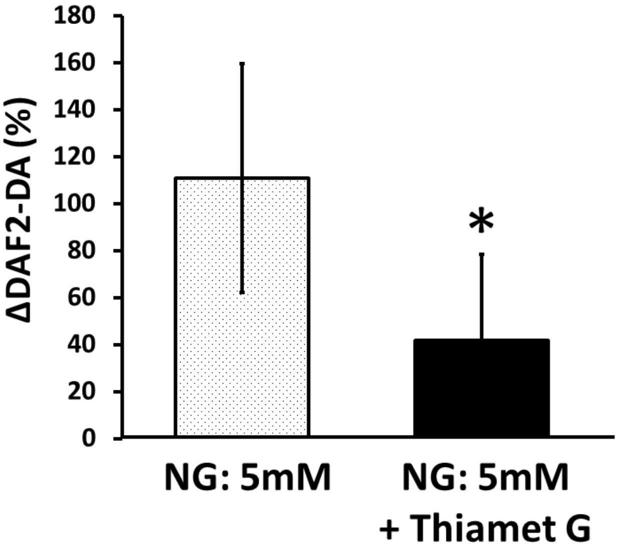


Arterial endothelial cells were freshly isolated and loaded with DAF-2 DA, treated with or without Thiamet G (1 μM, 8 hours). The fluorescence intensity was quantified. **A**. Representative cells treated with 0 or 100 nmol/L of insulin for 30 minutes are shown. **B**. The bar graph shows summed data on the reference of no treatment group (n = 8, P = 0.007). **C**. Insulin increased 110 ± 49% NO production in endothelial cells without treatment but 42 ± 37% in endothelial cells with incubation with Thiamet G (n = 8, P = 0.036).

DAF-2 DA (green), von Willebrand Factor (vWF) (red)

DAPI indicates 4′,6 ‑ diamidino ‑ 2 ‑ phenylindole stain (blue).

Data are expressed as mean ± standard error *: $p < 0.05$

**Figure S2. Increased O-GlcNAc modification by Thiamet G.**

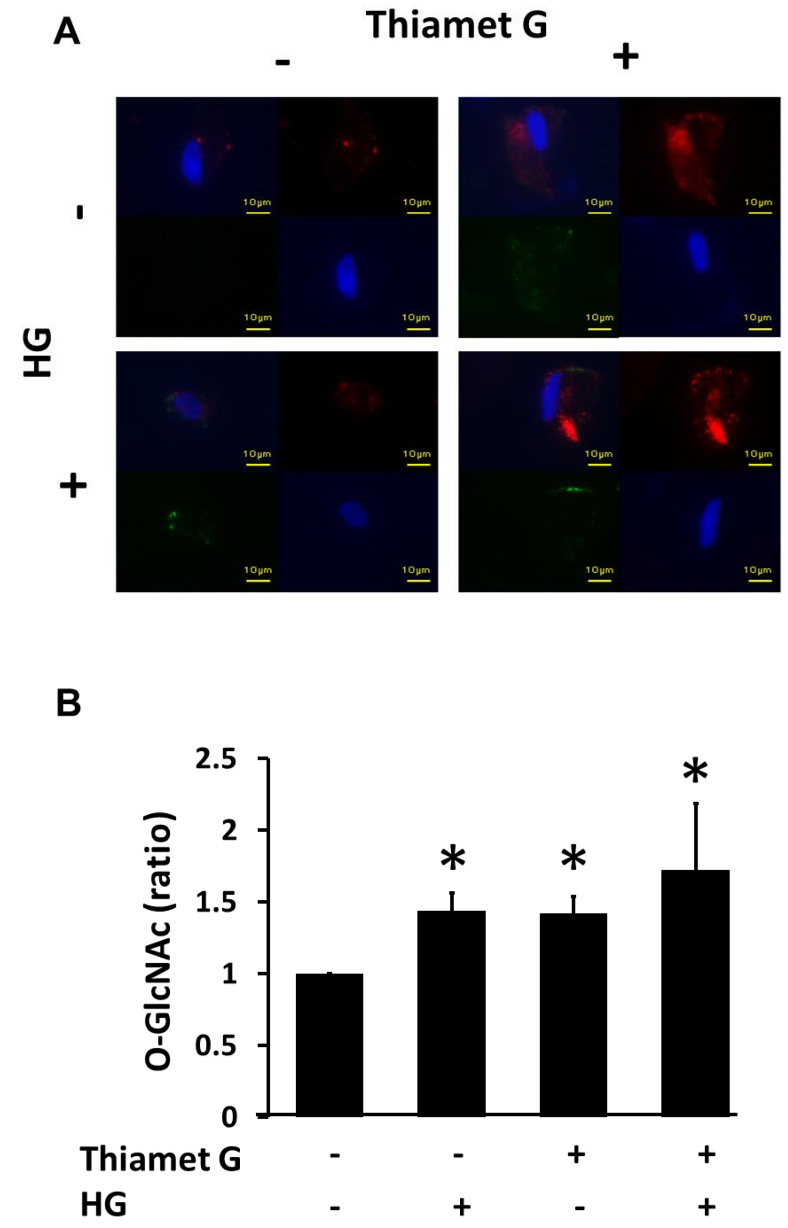


Arterial endothelial cells from nondiabetic patients were freshly isolated and incubated for 8 hours. The fluorescence intensity of O-GlcNAc modification was quantified. **A**. Representative cells treated with normal glucose condition (NG: 5

mmol/L), high-glucose condition (HG: 30mmol/L), NG with Thiamet G (1 μM), or HG with Thiamet G are shown. **B**. The bar graph shows summed data on the reference of no treatment group (HG: 1.44 ± 0.13, Thiamet G: 1.44 ± 0.11, HG + Thiamet G: 1.72 ± 0.46, n = 5, P = 0.022).

O-GlcNAc (RL2) (green), von Willebrand Factor (vWF) (red)

DAPI indicates 4′,6 ‐ diamidino ‐ 2 ‐ phenylindole stain (blue).

Data are expressed as mean ± standard error *: $p < 0.05$ vs. no treatment group
